# Blood and breath profiles of volatile organic compounds in patients with end-stage renal disease

**DOI:** 10.1186/1471-2369-15-43

**Published:** 2014-03-08

**Authors:** Paweł Mochalski, Julian King, Matthias Haas, Karl Unterkofler, Anton Amann, Gert Mayer

**Affiliations:** 1 Breath Research Institute, University of Innsbruck, Rathausplatz 4, A-6850 Dornbirn, Austria; 2 Institute of Nuclear Physics PAN, Radzikowskiego 152, PL-31342 Kraków, Poland; 3 Univ.-Clinic for Anesthesia, Innsbruck Medical University, Anichstrasse 35, A-6020 Innsbruck, Austria; 4 Department of Internal Medicine IV-Nephrology and Hypertension, Innsbruck Medical University, Anichstrasse 35, A-6020 Innsbruck, Austria

**Keywords:** Blood analysis, End-stage renal disease, Breath analysis, Hemodialysis, Uremic syndrome, Volatile organic compounds

## Abstract

**Background:**

Monitoring of volatile organic compounds (VOCs) in exhaled breath shows great potential as a non-invasive method for assessing hemodialysis efficiency. In this work we aim at identifying and quantifying of a wide range of VOCs characterizing uremic breath and blood, with a particular focus on species responding to the dialysis treatment.

**Methods:**

Gas chromatography with mass spectrometric detection coupled with solid-phase microextraction as pre-concentration method.

**Results:**

A total of 60 VOCs were reliably identified and quantified in blood and breath of CKD patients. Excluding contaminants, six compounds (isoprene, dimethyl sulfide, methyl propyl sulfide, allyl methyl sulfide, thiophene and benzene) changed their blood and breath levels during the hemodialysis treatment.

**Conclusions:**

Uremic breath and blood patterns were found to be notably affected by the contaminants from the extracorporeal circuits and hospital room air. Consequently, patient exposure to a wide spectrum of volatile species (hydrocarbons, aldehydes, ketones, aromatics, heterocyclic compounds) is expected during hemodialysis. Whereas highly volatile pollutants were relatively quickly removed from blood by exhalation, more soluble ones were retained and contributed to the uremic syndrome. At least two of the species observed (cyclohexanone and 2-propenal) are uremic toxins. Perhaps other volatile substances reported within this study may be toxic and have negative impact on human body functions. Further studies are required to investigate if VOCs responding to HD treatment could be used as markers for monitoring hemodialysis efficiency.

## Background

Chronic kidney disease (CKD) usually is a progressive disorder and patients with end stage renal failure need treatment by transplantation or dialysis. Even though dialysis is lifesaving, overall patient mortality by far exceeds that of an age-matched population without CKD. This is at least in part due to the fact that current dialysis techniques cannot completely replace native kidney function. Guidelines recommend that dialysis dose should be prescribed based on measures of urea clearance [1] even though it is recognized that urea is only a weak surrogate for the extent of uremia. However other, potentially more relevant toxins [2] cannot be determined easily due to technical difficulties and a rapid, low-cost measurement method, which could be used routinely during each HD session, would be highly desirable. Breath gas analysis could meet the requirements as it is non-invasive and breath biomarkers have already proved to provide valuable information on disease processes, or metabolic disorders occurring even in distant parts of the body [3-8]. Relying on simple-in-use, hand-held, and sensitive devices this diagnostic technique could considerably support the HD treatment.

The composition of uremic breath and its evolution during hemodialysis has already received some attention. Simenhoff *et al. *[9] reported elevated levels of dimethylamine (DMA) and trimethylamine (TMA) in patients with end-stage renal disease and their decrease after the HD treatment. In case of TMA this response to HD has been confirmed by Endre *et al. *[10]. A number of studies investigated breath ammonia during dialysis as a potential marker of efficiency of this treatment [10-14]. The observed drop in NH_3_ concentrations correlated reasonably well with blood urea nitrogen (BUN) and creatinine [12,14]. Monitoring of breath ethane in HD patients evidenced a rapid rise of this marker during the first minutes after initiation of dialysis, which has been attributed to treatment-induced oxidative stress [15-17]. Numerous investigators reported a significant increase of isoprene in breath during and after hemodialysis [18-22], but not during peritoneal dialysis [23]. Recently, Lee *et al. *[24] employing breath analysis documented the exposure of dialyzed populations to some hydrocarbons and halocarbons emitted by dialyzer materials.

The primary goal of this work was the untargeted identification and quantification of volatile organic compounds characterizing uremic breath and blood with a particular focus on species responding to the dialysis treatment, the latter being potential markers of dialysis efficiency. Gas chromatography with mass spectrometric detection using solid phase microextraction (SPME-GCMS) as a preconcentration step was selected as the adequate analytical method for this purpose. In particular, we aimed at extending the classic definition of uremic breath and blood (encompassing mainly odorous compounds such as ammonia or amines) to include a wider spectrum of volatiles, induced by either CKD or by the hemodialysis procedure itself.

## Methods

### Human subjects and HD treatment

Patients (7 males and 7 females) were recruited from the Dialyse Trainingszentrum Innsbruck and the Department of Nephrology and Hypertension of Innsbruck Medical University. Their baseline characteristics are presented in Table 1. All test subjects were dialyzed three times a week during 4-hours-sessions using high-flux Helixone Fresenius FX-100 dialyzers (Fresenius Medical Care, Germany) and a Fresenius type 5008, or Gambro type AK 200 S device. Patients suffered from a variety of underlying conditions including coronary heart disease, peripheral vascular disease, asthma, cancer, liver diseases and urinary tract infections. Four patients were diabetic. The recruitment was carried out with the following exclusions: inability to provide breath samples, immaturity and legal incompetence. No special dietary regimes were applied both before and during HD treatment. All subjects gave written consent to participate. The collection of samples was approved by the Ethics Commission of Innsbruck Medical University.

**Table 1:** Baseline characteristics of CKD patients under study

**Patient ID**	**Gender**	**Type of dialysis**	**Dialytic age**	**Dialysis efficiency**
	**[M/F]**	**[HD/HDF]**	**[months]**	**[Kt/V]**
193	M	HD	65	1.6*
195	F	HD	45	1.7*
202	M	HDF	31	1.5
203	F	HD	20	1.5
204	M	HD	13	0.8
205	F	HDF	10	2.2
206	M	HD	42	1.8
207	F	HDF	8	1.3
208	M	HD	47	1.4*
209	M	HDF	43	1.4
210	F	HD	37	1.1
211	F	HD	16	1.5*
213	F	HD	6	0.8
214	M	HD	13	1.5*

Patients’ mean age was 66 years (36-83).

*Not calculated on the sampling day.

### Identification of volatiles emitted by dialyzer materials

Contaminants emitted by the extracorporeal circuit materials and thus being introduced into the organism during the HD treatment were identified on the basis of analyses of air within freshly unpacked bloodlines and Helixone membranes as well as head-space above fragments of the aforementioned materials placed in sealed vials. In the latter case vials were stored for 1-2 hours to let potential pollutants accumulate.

### Blood and breath sampling

Patients were dialyzed via an upper extremity native fistula and provided two blood samples taken from the arterial inflow line into 2.7 mL blood monovettes (Sarstedt, Germany) using a protocol described in our recent article [25]. The first blood sample was taken shortly (<10 min) after the onset of dialysis procedure and the second one a few minutes before its end. In parallel, one blank sample containing 2.7 mL of distilled water was collected using the same materials as in the case of blood sampling. This was done to identify possible contaminants stemming from sources other than blood. Blank samples were analyzed in the same way as blood samples and the resulting concentration levels were subtracted from the respective blood sample values.

End-exhaled breath samples were collected into 3-liter Tedlar bags (SKC Inc., USA) in a CO_2_ controlled manner using breath sampler developed at Innsbruck Medical University, Austria [25-27]. The evolution of breath constituents during dialysis was monitored by taking breath samples every 40-60 minutes throughout the dialysis session. The first sampling instant was at the onset of hemodialysis, the last one just before removing the extracorporeal circuit. Effectively, 5-7 breath samples were collected per patient. Additionally, two separate room air samples were taken (one at the onset of dialysis and one at the end of the treatment) for determining the background levels of VOCs under study.

### Blood/breath sample preparation and chromatographic analysis

Blood sample preparation, breath and blood calibration and the chromatographic analysis itself were performed in analogy with the procedures outlined in our recent article [25]. However, breath sample preparation relied on solid phase microextraction (SPME) and followed procedure described in King *et al. *[28].

We stress the fact that the identification of compounds was performed in two steps. First, the peak spectrum was checked against the NIST mass spectral library. Next, the NIST identification was confirmed by comparing the retention times of peaks of interest with retention times obtained for standard mixtures prepared from pure compounds. Peak integration was based on extracted ion chromatograms. The applied quantifier ions are presented in Table 2.

**Table 2:** **Retention times R**_**t **_**[min], quantifier ions, LODs [nmol∙L**^**-1**^**, ppb], RSDs (%), coefficients of variation (R**^**2**^**) and linear ranges [nmol∙L**^**-1**^**, ppb] of compounds under study for blood and breath measurements**

**VOC**	**CAS**	**R**_**t**_	**Quantifier ion**	**Blood**				**Breath/room air**			
				**LOD**	**RSD**	**R**^**2**^	**Linear range**	**LOD**	**RSD**	**R**^**2**^	**Linear range**
		**[min]**		**[nmol/L]**	**[%]**		**[nmol/L]**	**[ppb]**	**[%]**		**[ppb]**
Propane, 2-methyl-	75-28-5	10.86	43	0.36	3.6	0.996	1.1-16	0.3	7	0.998	0.9-30
1,3-Butadiene	106-99-0	11.04	54	0.08	5	0.997	0.25-13	0.4	1.5	0.995	1.2-60
Acetonitrile	75-05-8	11.32	41	21	14	0.998	63-11000	4	8	0.999	11-150
n-Butane	106-97-8	11.78	43	0.13	3.5	0.999	0.4-16	0.2	4.5	0.988	0.6-100
2-Propenal	107-02-8	12.80	56	800	9	0.965	2500-25000	0.9	7	0.997	2.7-63
Furan	110-00-9	13.32	68	0.03	3	0.991	0.07-70	0.2	2	0.999	0.6-23
Propanal	123-38-6	13.42	58	1.5	5	0.991	4.5-1700	0.7	3	0.997	2-150
Acetone	67-64-1	13.56	58	-	-	-	-	0.8	3	0.999	2.4-10000
Dimethyl sulfide (DMS)	75-18-3	14.27	62	0.24	1.5	0.995	0.7-140	0.06	1.5	0.999	0.17-60
Butane, 2-methyl-	78-78-4	15.90	57	0.10	7	0.995	0.3-4	0.3	2	0.996	0.9-46
Isoprene	78-79-5	16.05	67	0.02	3.5	0.995	0.06-58	0.04	1.5	0.999	0.12-500
2-Pentene, (Z)-	627-20-3	16.41	55	0.02	6	0.989	0.06-3.5	0.15	2.5	0.998	0.45-20
n-Pentane	109-66-0	16.54	43	0.1	2.5	0.988	0.3-5	0.12	1.6	0.996	0.36-60
1,3-Pentadiene, (E)-	2004-70-8	16.75	67	0.02	3.4	0.999	0.06-2	0.07	1.5	0.999	0.2-20
1,3-Pentadiene, (Z)-	1574-41-0	16.86	67	0.02	4.5	0.997	0.06-1.5	0.07	2	0.998	0.2-13
2-Propenal, 2-methyl-	78-85-3	16.92	70	3	9	0.993	10-250	0.1	1	0.998	0.3-50
3-Buten-2-one	78-94-4	17.54	55	40	11	0.985	120-10000	0.2	7	0.998	0.6-23
Furan, 2-methyl-	534-22-5	18.05	82	0.02	3.5	0.993	0.06-6.5	0.08	2	0.998	0.24-18
2,3-Butanedione	431-03-8	18.06	86	10	13	0.986	30-4000	0.4	3.3	0.994	1-200
2-Butanone	78-93-3	18.11	43	0.4	6	0.999	1.3-3000	0.13	7	0.997	0.38-250
Furan, 3-methyl-	930-27-8	18.21	82	0.02	4	0.991	0.06-4	0.09	2	0.997	0.3-10
Ethyl Acetate	141-78-6	18.91	43	0.3	9	0.960	0.9-400	0.13	2.5	0.996	0.39-200
Thiophene	110-02-1	19.84	84	0.04	1	0.994	0.12-6.5	-	-	-	-
Pentane, 2-methyl-	107-83-5	19.95	43	0.3	1.5	0.993	0.9-5	0.17	1	0.999	0.53-150
1-Pentene, 2-methyl-	763-29-1	19.99	56	0.07	5.5	0.997	0.2-7	0.1	3.2	0.999	0.3-15
1-Hexene	592-41-6	20.17	56	0.03	5.5	0.997	0.09-10	0.2	1.7	0.999	0.6-70
Pentane, 3-methyl-	96-14-0	20.21	57	0.12	4	0.998	0.38-5	0.08	1.5	0.999	0.24-40
Benzene	71-43-2	20.32	78	0.04	3.2	0.995	0.12-8	0.11	3.8	0.998	0.33-36
n-Hexane	110-54-3	20.65	57	0.03	2.5	0.993	0.1-8	0.12	1.6	0.995	0.36-110
Pyrrole	109-97-7	20.81	67	0.5	14	0.982	1.5-100	-	-	-	-
Cyclohexane	110-82-7	21.33	56	0.07	3	0.996	0.21-6	0.1	6	0.997	0.3-100
Pyrimidine	289-95-2	21.64	80	7	12	0.990	20-950	-	-	-	-
2-Pentanone	107-87-9	21.88	43	1	8	0.975	3-1000	0.08	2.2	0.998	0.24-24
Furan, 2,5-dimethyl-	625-86-5	22.00	96	0.08	4	0.995	0.24-5.5	0.08	1.4	0.999	0.24-15
Sulfide, allyl methyl (AMS)	10152-76-8	22.05	88	0.1	2	0.998	0.3-15	0.14	2	0.999	0.43-15
Pyridine	110-86-1	22.05	79	0.6	9	0.988	2-450	0.04	9	0.975	0.12-100
Sulfide, methyl propyl (MPS)	3877-15-4	22.67	61	0.12	1.5	0.999	0.36-50	0.04	2.1	0.996	0.12-30
Hexane, 2-methyl-	591-76-4	23.60	85	0.07	3	0.990	0.2-4.5	0.09	3	0.998	0.26-11
1-Heptene	592-76-7	23.87	56	0.09	5	0.998	0.27-4.2	0.13	2	0.999	0.38-70
2-Heptene, (E)-	592-77-8	24.09	55	0.07	6	0.996	0.21-5.3	0.4	1.4	0.994	1.2-13
Toluene	108-88-3	24.26	91	0.02	7	0.998	0.06-15	0.05	3	0.993	0.15-30
n-Heptane	142-82-5	24.30	43	0.13	2	0.997	0.4-4	0.13	1.5	0.998	0.39-40
2-Hexanone	591-78-6	25.49	58	0.1	9	0.997	0.3-25	0.18	1	0.989	0.54-13
Heptane, 3-methylene-	1632-16-2	26.91	70	0.03	3	0.998	0.09-45	0.09	1	0.994	0.26-20
3-Octene, (E)-	14919-01-8	27.25	55	0.07	4	0.991	0.2-20	0.32	2	0.991	1-13
Cyclohexanone	108-94-1	27.41	55	1.3	13	0.999	4-700	0.12	8	0.956	0.34-80
p-Xylene	106-42-3	27.68	91	0.03	8	0.998	0.09-10	0.07	8	0.986	0.2-18
2-Cyclohexen-1-one	930-68-7	27.89	68	2	12	0.998	6-460	0.1	10	0.981	0.3-15
o-Xylene	95-47-6	27.95	91	0.03	7	0.998	0.09-11	0.08	9	0.991	0.24-10
4-Heptanone	123-19-3	28.29	71	0.17	9	0.997	0.5-430	0.05	6.3	0.978	0.16-17
3-Heptanone	106-35-4	28.51	57	0.44	11	0.998	1.3-350	0.2	5	0.977	0.6-6
2-Heptanone	110-43-0	28.68	43	0.6	10	0.997	1.8-320	0.1	6.3	0.995	0.3-9
Heptane, 2,4-dimethyl-	2213-23-2	92.01	85	0.06	6	0.975	0.2-3	0.1	6	0.987	0.3-9
α-Pinene	80-56-8	30.69	93	2	9	0.986	6-440	0.46	8	0.985	1.4-20
Furan, 2-pentyl-	3777-69-3	31.53	81	3.5	13	0.989	10-350	-	-	-	-
3-Carene	13466-78-9	32.09	93	1.4	8	0.997	4.2-280	0.6	5.3	0.954	1.8-12
m-Cymene	535-77-3	32.34	119	0.6	9	0.992	1.8-90	0.1	5.5	0.981	0.3-10
p-Cymene	99-87-6	32.62	119	1	9.5	0.993	3-160	0.1	6	0.973	0.3-21
DL-Limonene	138-86-3	32.84	68	1	9	0.983	3-1000	0.46	6	0.954	1.4-30
Eucalyptol	470-82-6	33.40	43	0.7	14	0.989	2-3	-	-	-	-

Compounds are ordered with respect to increasing retention time.

## Results and discussion

### Method validation

Limits of detection/quantification (LOD/LOQ) as well as relative standard deviations (RSDs) for the measurements were calculated as described in [25]. The obtained validation parameters are shown in Table 2 and were recognized as satisfactory for the goals of this study. With a few exceptions (pyrrole, pyrimidine and 2-pentyl-furan), most of the reported compounds could be quantified in both blood and breath. Blood acetone levels generally exceeded the dynamic range of the MS detector.

### Contaminants emitted by dialyzer materials

The Helixone membrane was found to emit predominantly n-alkanes (C3-C7), methylated alkanes (2-methyl-propane, 2-methyl-butane, 2-methyl-pentane and 2,4-dimethyl-heptane) and alkenes (propene, 2-methyl-1-propene, 2-pentene, 1-hexene and 2,4-dimethyl-1-heptene). Apart from hydrocarbons it also emitted considerable amounts of acetone and hexamethyl disiloxane.

More than 60 volatile contaminants were found to be released by the bloodlines and 43 of them could reliably be identified. Among these, hydrocarbons were the predominant chemical class with eighteen representatives. This number includes n-alkanes (C4-C7), methylated alkanes (2-methyl-propane, 2-methyl-pentane, 3-methyl-pentane, 4-methyl-heptane, 4-methyl-octane), cyclic HCs (methyl cyclopentane and cyclohexane) and alkenes (2-methyl-1-propene, 1-pentene, 2-heptene, 3-heptene, 3-ethyl-3-hexene, 3-methylene-heptane, 4-octene and 2,4-dimethyl-1-heptene). Furthermore, there were nine aldehydes (2-propenal, propanal, 2-methyl-2-propenal, pentanal, hexanal, heptanal, benzaldehyde, 2-ethyl-hexanal and nonanal), seven ketones (acetone, 3-buten-2-one, 2-hexanone, cyclohexanone, 2-cyclohexen-1-one, 3-methyl-2-hexanone and 3-heptanone), three furans (furan, 2,3-dihydroxy-furan and tetrahydrofuran), three terpenes (α-pinene, β-pinene and 3-carene) and two aromatics (toluene and p-xylene). The only alcohol present was 2-ethyl-hexanol. Two species namely tetrahydrofuran and 2-ethyl-hexanol exhibited especially high abundances. Pollutants affecting the levels of species reported within this study have been marked in Table 3.

**Table 3:** **Detection (n**_**d**_**) and quantification (n**_**q**_**) incidences and concentration ranges of blood volatile organic compounds**

**Class**	**VOC**	**CAS**	**Blood before HD**		**Blood after HD**		**Wilcoxon blood before/after**	**Contaminants**	
			**Incidence**	**Range (median)**	**Incidence**	**Range (median)**		**Tubing**	**Membrane**
			**n**_**d**_**(n**_**q**_**)**	**[nmol/L]**	**n**_**d**_**(n**_**q**_**)**	**[nmol/L]**			
VSC	Dimethyl sulfide (DMS)	75-18-3	14(14)	2.3-146(18.7)	14(14)	1.2-38.7(5.7)	<0.001		
	Thiophene	110-02-1	8(6)	0.11-0.66(0.13)	2(1)	0.36	0.03		
	Sulfide, allyl methyl (AMS)	10152-76-8	8(8)	0.6-13(3.3)	8(6)	0.67-3.7(2.4)	0.02		
	Sulfide, methyl propyl (MPS)	3877-15-4	11(8)	0.7-56(4.3)	10(7)	0.4-17.5(1.1)	0.008		
Ketones	Acetone	67-64-1	14(14)	n.q.	14(14)	n.q.	-	●	●
	3-Buten-2-one	78-94-4	7(7)	488-7370(1090)	10(10)	385-47360(1170)	n.s.	●	
	2,3-Butanedione	431-03-8	1(1)	967	1(1)	285	-		
	2-Butanone	78-93-3	14(14)	13-1895(60)	14(14)	32-4330(164)	n.s.		
	2-Pentanone	107-87-9	14(14)	11.5-387(68)	14(14)	31.3-341(77)	n.s.		
	2-Hexanone	591-78-6	4(2)	2.4-3.2(2.8)	7(5)	0.4-6.8(0.75)	n.s.	●	
	Cyclohexanone	108-94-1	8(8)	57-478(238)	8(8)	11-780(150)	n.s.	●	
	2-Cyclohexen-1-one	930-68-7	4(4)	73-344(232)	4(4)	55-225(98.5)	-	●	
	4-Heptanone	123-19-3	14(14)	1.8-130(35)	14(14)	14.5-200(41)	0.03		
	3-Heptanone	106-35-4	8(8)	1.5-148(5)	9(9)	1.5-87(2.6)	n.s.	●	
	2-Heptanone	110-43-0	9(9)	1.5-31(6)	12(12)	3.5-128(19)	<0.001		
Ald.	2-Propenal	107-02-8	8(8)	4080-26100(12800)	3(3)	10200-15200(14800)	n.s.	●	
	Propanal*	123-38-6	7(7)	5.9-44(17.5)	5(5)	6.5-820(23)	n.s.	●	
	2-Propenal, 2-methyl-	78-85-3	1(1)	54	1(1)	54	-	●	
Hydrocarbons	Propane, 2-methyl-	75-28-5	1(1)	1.1	0(0)	<LOD	-	●	●
	1,3-Butadiene	106-99-0	2(2)	0.57-0.9(0.74)	1(1)	0.27	-		
	n-Butane	106-97-8	7(7)	0.5-2.3(1.25)	5(5)	0.4-5.6(1.2)	n.s.	●	●
	Butane, 2-methyl-	78-78-4	7(7)	0.21-2.2(0.57)	2(2)	0.12-0.46(0.29)	n.s.		●
	Isoprene	78-79-5	14(14)	1.3-25.6(4.1)	14(14)	2.8-36(6.6)	<0.001		
	2-Pentene, (Z)-	627-20-3	2(2)	0.13-0.6(0.37)	2(2)	0.1-0.27(0.19)	-		●
	n-Pentane	109-66-0	6(6)	0.35-2(0.67)	7(7)	0.3-2.7(1.1)	n.s.	●	●
	1,3-Pentadiene, (E)-	2004-70-8	2(2)	0.6-0.9(0.74)	1(1)	0.28	-		
	1,3-Pentadiene, (Z)-	1574-41-0	2(2)	0.17-0.18(0.17)	1(1)	0.07	-		
	1-Pentene, 2-methyl-	763-29-1	0(0)	<LOD	0(0)	<LOD	-	●	
	Pentane, 2-methyl-	107-83-5	5(4)	1.1-4.8(2.4)	5(3)	1.1-2.5(2.1)	n.s.	●	●
	Cyclohexane	110-82-7	1(1)	0.27	0(0)	<LOD	-	●	●
	1-Hexene	592-41-6	4(4)	0.15-8.7(3.3)	5(5)	0.09-6.1(0.4)	-		●
	Pentane, 3-methyl-	96-14-0	2(0)	<LOQ	2(0)	<LOQ	-	●	
	n-Hexane	110-54-3	10(10)	0.09-7.3(0.35)	9(9)	0.16-2.9(0.33)	n.s.	●	●
	Hexane, 2-methyl-	591-76-4	4(4)	0.22-0.83(0.28)	4(4)	0.20-0.4(0.24)	-		●
	1-Heptene	592-76-7	2(2)	0.28-0.63(0.46)	1(1)	1.8	-		
	2-Heptene	592-77-8	1(1)	3.8	1(1)	2.7	-	●	
	n-Heptane	142-82-5	8(8)	0.36-2(0.6)	7(7)	0.4-2.3(0.6)	n.s.	●	●
	Heptane, 3-methylene-	1632-16-2	9(9)	0.09-52(0.62)	10(10)	0.26-44(0.95)	n.s.	●	
	3-Octene, (E)-	14919-01-8	5(5)	0.2-16(0.75)	5(5)	0.3-12(1.6)	-	●	
	Heptane, 2,4-dimethyl-	2213-23-2	0(0)	<LOD	0(0)	<LOD	-		●
Terpenes	α-Pinene	80-56-8	6(6)	13.7-239(48)	6(6)	13.4-183(35.5)	0.03	●	
	3-Carene	13466-78-9	12(12)	6.2-116(12.5)	12(11)	4-65(11.6)	n.s.	●	
	m-Cymene	535-77-3	1(1)	2.33	1(1)	2	-		
	p-Cymene	99-87-6	14(14)	3.4-84.5(17)	14(14)	3.6-69.5(11.7)	n.s.		
	DL-Limonene	138-86-3	13(13)	22.6-982(163)	13(13)	23.7-1210(109)	n.s.		
	Eucalyptol	470-82-6	2(2)	126-310(218)	2(2)	79-173(126)	-		
Aromatics	Benzene	71-43-2	10(10)	0.14-7.1(1)	5(5)	0.19-2.33(1.3)	0.01		
	Toluene	108-88-3	11(11)	0.28-14(1.2)	10(10)	0.22-9.5(0.65)	0.01	●	
	p-Xylene	106-42-3	13(13)	0.36-9.4(0.9)	11(11)	0.44-6.2(1.2)	n.s.	●	
	o-Xylene	95-47-6	2(2)	0.39-2.7(1.54)	2(2)	0.17-0.24(0.21)	-		
Heterocyclic	Furan	110-00-9	7(7)	0.06-37(0.7)	9(9)	0.21-113(1.7)	0.03	●	
	Furan, 2-methyl-	534-22-5	5(5)	0.18-6.3(0.7)	5(5)	0.1-1.7(0.6)	-		
	Furan, 3-methyl-	930-27-8	4(4)	0.07-0.37(0.08)	4(4)	0.06-0.16(0.07)	-		
	Pyrrole	109-97-7	2(2)	16-20.8(18.4)	0(0)	<LOD	-		
	Pyrimidine	289-95-2	9(8)	15.9-485(45)	8(8)	22.4-166(44.5)	n.s.		
	Furan, 2,5-dimethyl-	625-86-5	3(3)	0.25-2.6(2.16)	3(3)	0.25-2.45(1.29)	-		
	Pyridine	110-86-1	10(10)	11.4-359(28.6)	8(8)	7.2-47(22.6)	n.s.		
	Furan, 2-pentyl-	3777-69-3	5(4)	11-68(13)	5(3)	10-34(13)	n.s.		
Other	Acetonitrile	75-05-8	6(6)	201-6790(835)	4(4)	180-2280(1000)	-		
	Ethyl Acetate	141-78-6	2(2)	0.9-916(458)	2(2)	25.4-53(39)	-		

n.s. – not significant. Isoprene increases in concentration during dialysis, whereas dimethyl sulfide (DMS), methyl propyl sulfide (MPS) and benzene decrease in concentration during dialysis. *Due to some technical problems breath profiles of one patient were excluded from the data analysis.

### Blood and breath profiles of volatiles during HD

Altogether 60 compounds were quantified in blood and breath of patients undergoing HD. Their detection and quantification incidences as well as the observed concentrations are given in Tables 3 and 4. In blood hydrocarbons comprised 32% of all quantified species, ketones 20%, heterocyclic compounds 14%, terpenes 11%, aromatics 7%, volatile sulphur compounds 7% and aldehydes 5%. In breath the predominant chemical classes were hydrocarbons (40%) and ketones (21%). Other well represented classes were heterocyclic compounds (10%), terpenes (8%), volatile sulphur compounds (6%), aromatics (6%) and aldehydes (6%).

**Table 4:** **Detection (n**_**d**_**) and quantification (n**_**q**_**) incidences and concentration ranges of breath and room air compounds**

**Class**	**VOC**	**Breath before HD**		**Room air before HD**		**Breath after HD**		**Room air after HD**		**Wilcoxon breath before/**	**Wilcoxon breath/room air**	**Wilcoxon breath/room air**
		**Incidence**	**Range (median)**	**Incidence**	**Range (median)**	**Breath after incidence**	**Before range (median)**	**After incidence**	**Range (median)**			
		**n**_**d**_**(n**_**q**_**)**	**[ppb]**	**n**_**d**_**(n**_**q**_**)**	**[ppb]**	**n**_**d**_**(n**_**q**_**)**	**[ppb]**	**n**_**d**_**(n**_**q**_**)**	**[ppb]**			
VSC	Dimethyl sulfide (DMS)	13(13)	1.1-58.3(7.8)	13(12)	0.35-2.9(0.85)	13(13)	1.1-31(6.5)	13(12)	0.18-1.7(0.75)	<0.001	<0.001	<0.001
	Thiophene	0(0)	<LOD	0(0)	<LOD	0(0)	<LOD	0(0)	<LOD	-	-	-
	Sulfide, allyl methyl (AMS)	7(6)	0.66-4.9(1.2)	0(0)	<LOD	7(5)	0.7-4.8(1.7)	0(0)	<LOD	n.s.	0.03	0.03
	Sulfide, methyl propyl (MPS)	7(7)	0.3-12.5(1.5)	7(6)	0.14-0.82(0.2)	7(7)	0.33-12(1.5)	6(4)	0.15-0.36(0.24)	0.03	0.02	0.02
Ketones	Acetone	13(13)	760-9070(1600)	13(13)	106-850(240)	13(13)	580-3900(1500)	13(13)	100-350(200)	n.s.	<0.001	<0.001
	3-Buten-2-one	12(12)	2.7-6.5(4.3)	12(12)	2-6.4(3.2)	12(12)	3.2-8.1(4.9)	12(12)	1.9-10.5(2.9)	n.s.	0.005	0.01
	2-Butanone	13(13)	2.7-225(19)	13(13)	2.9-140(43)	13(13)	2-49(12.6)	13(13)	2-67(12.5)	0.03	n.s.	n.s.
	2,3-Butanedione	12(12)	4.6-73(23.5)	12(12)	2.8-7.7(6.1)	12(12)	4.5-142(18)	12(12)	2.5-10.4(5.3)	n.s.	<0.001	<0.001
	2-Pentanone	13(13)	0.43-16.2(1.2)	13(10)	0.25-0.83(0.4)	13(13)	0.64-6.2(1.3)	13(10)	0.26-1(0.44)	n.s.	<0.001	<0.001
	2-Hexanone	1(1)	2.07	1(1)	0.56	1(1)	0.61	1(1)	0.59	-	-	-
	Cyclohexanone	9(9)	0.76-43(18)	8(8)	1.2-118(32)	8(8)	0.77-21.3(11.6)	8(8)	0.92-70(32.3)	n.s.	0.02	0.01
	2-Cyclohexen-1-one	6(6)	0.7-5(2)	6(6)	2.1-12(3)	5(4)	1-2.5(1.8)	5(5)	1.3-5(3.5)	-	-	-
	4-Heptanone	10(10)	0.17-1.35(0.5)	1(0)	<LOD	10(9)	0.21-1.5(0.55)	1(0)	<LOD	n.s.	0.003	0.004
	3-Heptanone	7(2)	0.79-0.85(0.82)	7(1)	0.64	7(0)	<LOQ	7(0)	<LOQ	-	-	-
	2-Heptanone	2(2)	0.45-0.96(0.7)	2(2)	0.34-0.4(0.37)	2(1)	0.62	2(2)	0.36-0.4(0.38)	-	-	-
Ald.	2-Propenal	12(12)	10-51(22)	12(12)	8.7-47(20.6)	12(12)	9-57(21.8)	12(12)	9.6-50(21.3)	n.s.	n.s.	n.s.
	Propanal^*^	12(12)	6.4-80(18)	12(12)	15.7-227(49)	12(12)	4.7-37.3(11.7)	12(12)	11.7-74.4(34.4)	0.005	<0.001	<0.001
	2-Propenal, 2-methyl-	12(12)	2.8-5.8(4.2)	12(12)	2.9-14(3.4)	12(12)	2.2-15(4.8)	12(12)	1.7-22(3.7)	n.s.	n.s.	n.s.
Hydrocarbons	1,3-Butadiene	3(3)	5.6-18(10)	3(3)	2-3.5(2.9)	3(3)	3.7-7(5)	3(3)	1.8-3(2.5)	-	-	-
	Propane, 2-methyl-	4(4)	8.6-24.4(17)	4(4)	2.14-23.7(5.5)	4(4)	6.7-20(13.4)	4(4)	2.74-18.9(5.4)	n.s.	n.s.	n.s.
	n-Butane	12(12)	8.1-147(19.4)	12(12)	2.75-21.4(7.9)	12(12)	3.1-111(12.9)	12(12)	2.66-19.2(7.2)	<0.001	0.002	n.s.
	Butane, 2-methyl-	12(12)	7-27.3(13.6)	12(12)	3.9-32(11.4)	12(12)	4.8-26(9.4)	12(12)	4.4-26(11.5)	n.s.	n.s.	n.s.
	Isoprene	13(13)	50-563(101)	13(13)	1.9-7(3)	13(13)	116-547(199)	13(13)	2.4-6.6(4.4)	0.009	<0.001	<0.001
	2-Pentene, (Z)-	4(4)	2.5-17(14.7)	4(4)	1.1-8.6(5.2)	4(4)	0.97-10.5(7)	4(4)	0.7-15.6(8.2)	n.s.	n.s.	n.s.
	n-Pentane	13(13)	9.3-70(16.9)	13(13)	2.1-14.9(6)	13(13)	2.07-14.4(4.8)	13(13)	1.48-11.9(2.9)	<0.001	<0.001	<0.001
	1,3-Pentadiene, (E)-	3(3)	5.6-18.1(11.3)	3(3)	2-3.5(2.8)	3(3)	3.7-7(5.3)	3(3)	1.79-3.1(2.44)	-	-	-
	1,3-Pentadiene, (Z)-	3(3)	2.6-6.9(4.3)	3(3)	0.37-1(0.67)	3(3)	1.1-2.9(1.9)	3(3)	0.36-0.8(0.59)	-	-	-
	1-Pentene, 2-methyl-	7(7)	1.43-15.8(2.7)	7(4)	0.3-1.3(0.55)	7(4)	0.44-1.77(0.9)	6(2)	0.8-0.89(0.85)	0.02	0.02	n.s.
	Pentane, 2-methyl-	6(6)	9.4-130(41)	6(6)	9.2-171(36.4)	6(6)	2.3-31(23.3)	6(6)	2.7-45(14.6)	0.03	n.s.	n.s.
	Cyclohexane	11(11)	0.99-3.4(2.2)	11(11)	0.53-113(1.5)	11(11)	0.57-123(2.1)	11(11)	0.4-95(1.7)	n.s.	n.s.	n.s.
	Hexane, 2-methyl-	9(9)	0.47-4.8(0.84)	9(8)	0.71-2.8(0.9)	9(8)	0.56-3.2(1.3)	9(8)	0.51-2.5(0.86)	n.s.	n.s.	0.008
	1-Hexene	9(9)	1.32-77(5.4)	9(9)	0.93-77(5.5)	9(9)	0.79-20(4.5)	9(9)	0.93-16.6(3.7)	0.04	0.03	n.s.
	Pentane, 3-methyl-	8(8)	2.5-32.5(7.3)	8(8)	3.1-42(7.5)	8(8)	0.93-8.4(4.4)	8(8)	0.94-11.4(2.9)	0.008	n.s.	n.s.
	n-Hexane	13(13)	1.4-21(4.4)	13(13)	1-13.7(4.6)	13(13)	1.2-121(5.8)	13(13)	0.9-35(3.9)	n.s.	n.s.	n.s.
	1-Heptene	8(8)	0.57-56(1.4)	8(8)	0.47-5(0.77)	8(8)	0.46-7.3(1.6)	8(8)	0.39-3.8(0.9)	n.s.	0.02	0.04
	2-Heptene	0(0)	<LOD	0(0)	<LOD	0(0)	<LOD	0(0)	<LOD	-	-	-
	n-Heptane	13(13)	0.91-33.4(1.9)	13(13)	0.62-2.8(1.4)	13(13)	0.69-3.3(1.7)	13(13)	0.53-3.5(1.3)	<0.001	<0.001	0.02
	Heptane, 3-methylene-	13(13)	0.95-17.5(2.6)	13(11)	0.28-1.3(0.52)	13(13)	0.5-9.6(1.9)	13(11)	0.33-2.4(0.57)	n.s.	<0.001	<0.001
	3-Octene, (E)-	11(8)	1.7-4.7(2.2)	11(4)	0.98-1.7(1.3)	11(8)	0.98-2.5(1.9)	11(4)	1-2.4(1.6)	n.s.	0.008	n.s.
	Heptane, 2,4-dimethyl-	0(0)	<LOD	0(0)	<LOD	0(0)	<LOD	0(0)	<LOD	-	-	-
Terpenes	α-Pinene	12(11)	1.94-16.3(2.8)	12(7)	1.6-7.2(2.1)	12(10)	1.85-7.2(2.4)	12(4)	1.7-3.53(2.7)	n.s.	n.s.	n.s.
	3-Carene	2(1)	7.1	1(0)	<LOQ	2(1)	2.53	2(0)	<LOQ	n.s.	n.s.	n.s.
	m-Cymene	0(0)	<LOD	0(0)	<LOD	0(0)	<LOD	0(0)	<LOD	-	-	-
	p-Cymene	13(13)	0.36-3.7(0.97)	13(13)	0.3-1.58(0.77)	13(13)	0.29-3.1(0.86)	13(13)	0.29-2.15(0.76)	0.02	0.04	0.05
	DL-Limonene	13(13)	1.6-22(5.1)	13(13)	1.7-7.3(4)	13(13)	1.7-19.7(4.4)	13(13)	1.7-6.9(4.3)	n.s.	n.s.	n.s.
	Eucalyptol	0(0)	<LOD	0(0)	<LOD	0(0)	<LOD	0(0)	<LOD	-	-	-
Aromatics	Benzene	13(13)	2.1-15(4.2)	13(13)	1.91-6.7(3.8)	13(13)	1.9-8.7(4.0)	13(13)	1.7-8.1(3.5)	0.002	<0.001	n.s.
	Toluene	13(13)	2.2-15.2(5.1)	13(13)	2.12-9.4(4)	13(13)	2-10.2(4.3)	13(13)	2.14-9.9(4.2)	0.02	0.008	n.s.
	p-Xylene	12(12)	2.8-7.7(4.3)	12(12)	2.9-7.9(4.4)	12(12)	3-8.5(4.2)	12(12)	3.3-7.8(4.3)	n.s.	n.s.	n.s.
	o-Xylene	0(0)	<LOD	0(0)	<LOD	0(0)	<LOD	0(0)	<LOD	-	-	-
Heterocyclic	Furan	11(11)	0.68-9.8(2.0)	11(9)	0.7-2.2(1.5)	11(9)	0.85-4.1(1.8)	11(9)	0.65-2.9(1.3)	<0.001	<0.001	n.s.
	Furan, 2-methyl-	13(13)	0.52-7.1(1.32)	13(13)	0.47-1.77(0.68)	13(13)	0.59-3.24(1)	13(13)	0.52-1.88(0.84)	n.s.	0.04	0.03
	Furan, 3-methyl-	13(8)	0.3-1.1(0.43)	13(2)	0.35-0.42(0.38)	13(8)	0.32-1.1(0.41)	13(3)	0.39-0.5(0.4)	n.s.	<0.001	<0.001
	Furan, 2,5-dimethyl-	2(2)	0.58-2.8(1.7)	2(0)	<LOQ	2(1)	1.13	2(0)	<LOQ	-	-	-
	Pyridine	13(13)	0.75-58(7.3)	13(13)	0.4-75(5.9)	13(13)	0.66-141(11)	13(13)	0.4-74(15.3)	n.s.	n.s.	n.s.
	Furan, 2-pentyl-	0(0)	<LOD	0(0)	<LOD	0(0)	<LOD	0(0)	<LOD	-	-	-
Other	Acetonitrile	13(11)	12-165(15.4)	13(8)	11.5-46.5(14.5)	13(8)	13-56(16.8)	13(9)	12-55(15.1)	n.s.	n.s.	n.s.
	Ethyl Acetate	13(13)	0.8-211(1.3)	13(13)	0.9-47.7(1.5)	13(13)	0.68-48(1.04)	13(13)	0.98-45.7(1.4)	n.s.	n.s.	n.s.

n.s. – not significant.

^*^Due to some technical problems breath profiles of one patient were excluded from the data analysis.

**Figure 1:**
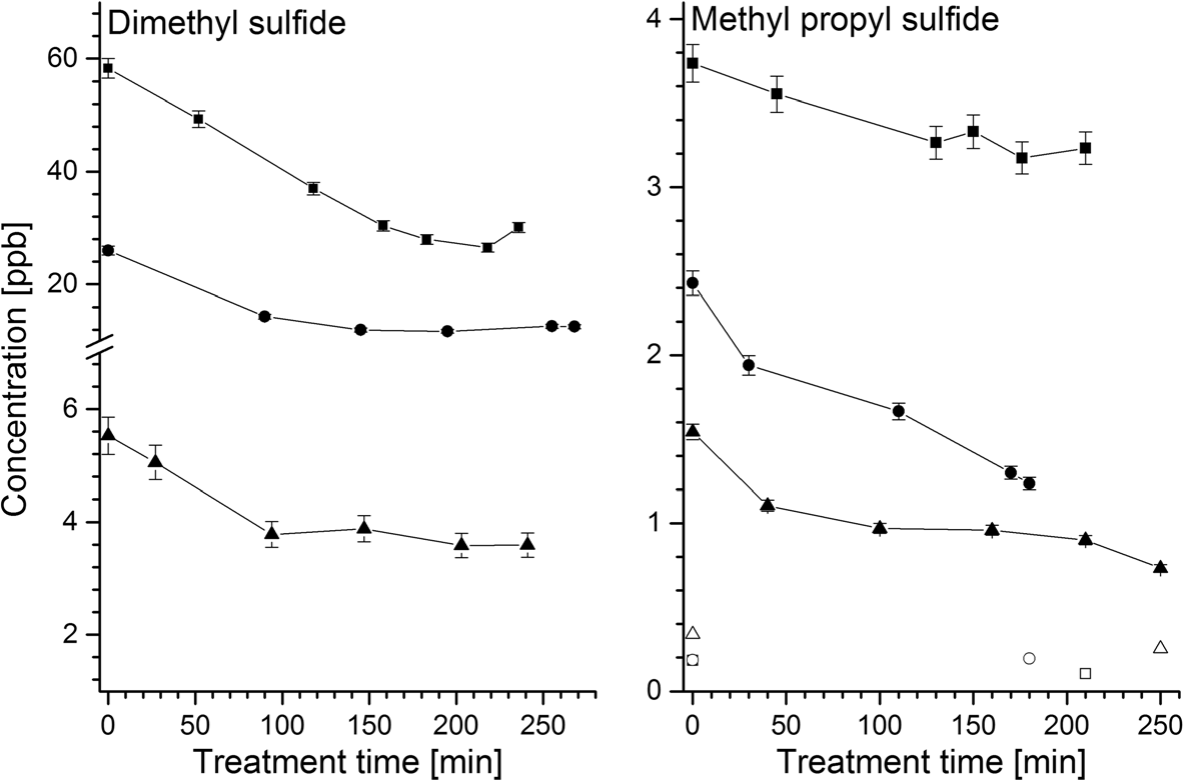
**Representative breath profiles of MPS and DMS during hemodialysis.** Hollow and filled symbols denote room air and breath levels respectively.

Four volatile sulphur compounds (DMS, AMS, MPS and thiophene) were quantified in blood and breath. Interestingly, their concentrations were decreasing during the HD treatment in both investigated fluids (see Tables 3 and 4 for Wilcoxon tests and Figure 1 for representative MPS and DMS profiles). For example, blood levels of DMS after the HD session were on average three-fold lower than at the onset of the treatment. Analogously, breath concentrations dropped approximately by a factor of 1.5. The reason for this response of sulphur species remains unclear. They were not found to be released by the dialyzer materials and their room air levels were relatively low. Thus, exogenous contamination can be excluded as a potential reason for elevated VSC concentrations before HD. It rather seems that volatile sulphur species were effectively removed from blood during the haemodialysis session. Conversely, Goerl *et al*. [29] reported stable levels of DMS in breath of patients undergoing dialysis. The reason for this discrepancy remains speculative. Perhaps, some differences in HD treatment affect DMS washout.

**Figure 2:**
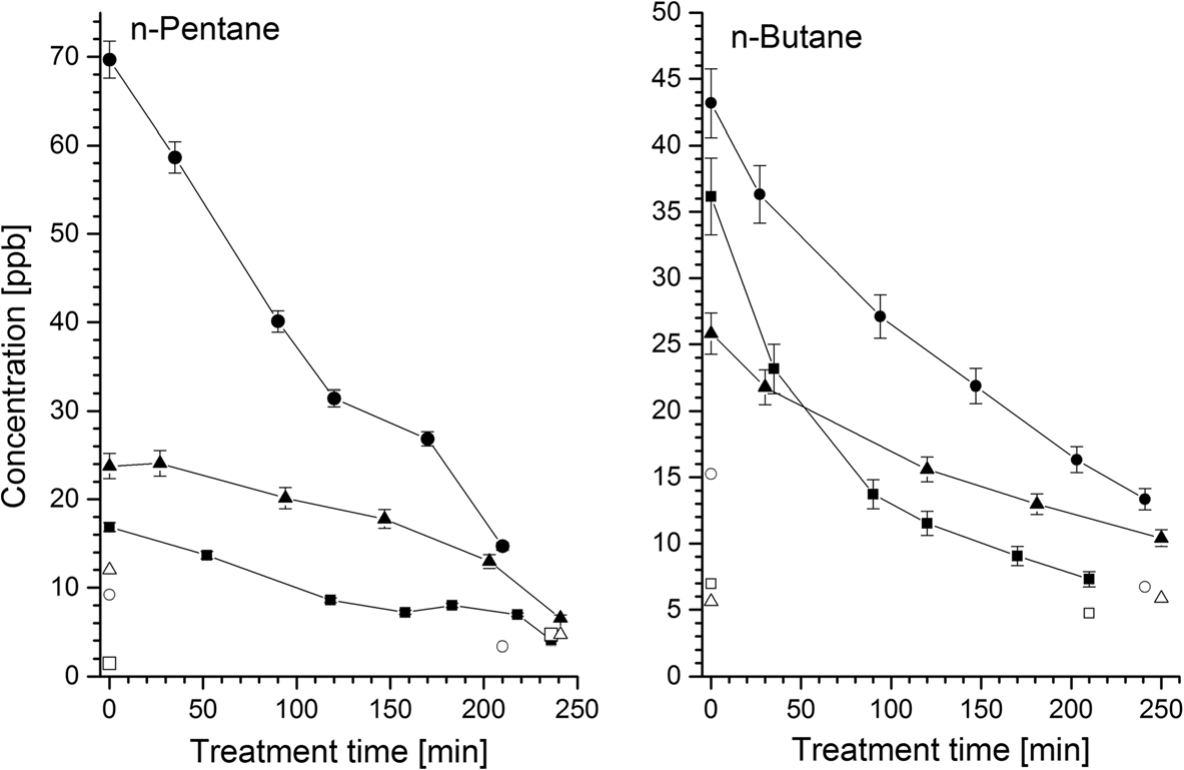
**Representative breath profiles of n-butane and n-pentane during hemodialysis.** Hollow and filled symbols denote room air and breath levels respectively.

**Figure 3:**
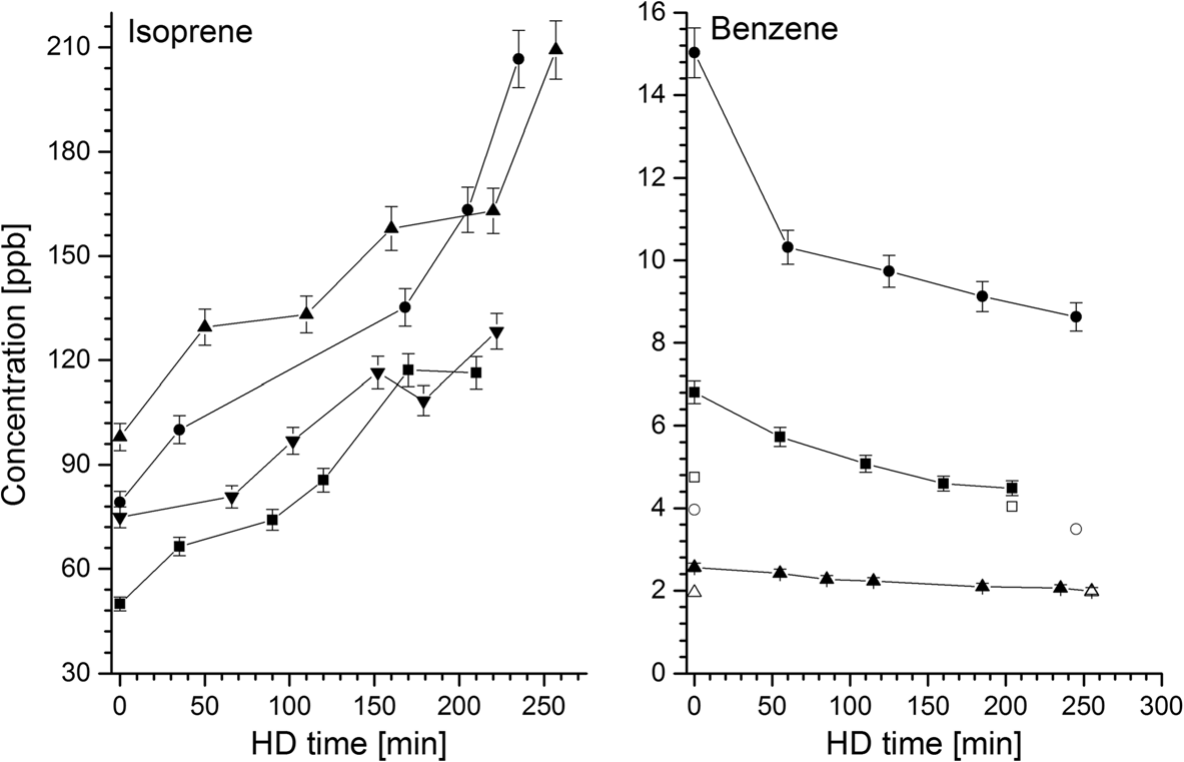
**Representative breath profiles of benzene, and isoprene during hemodialysis.** Hollow and filled symbols denote room air and breath levels respectively.

**Figure 4:**
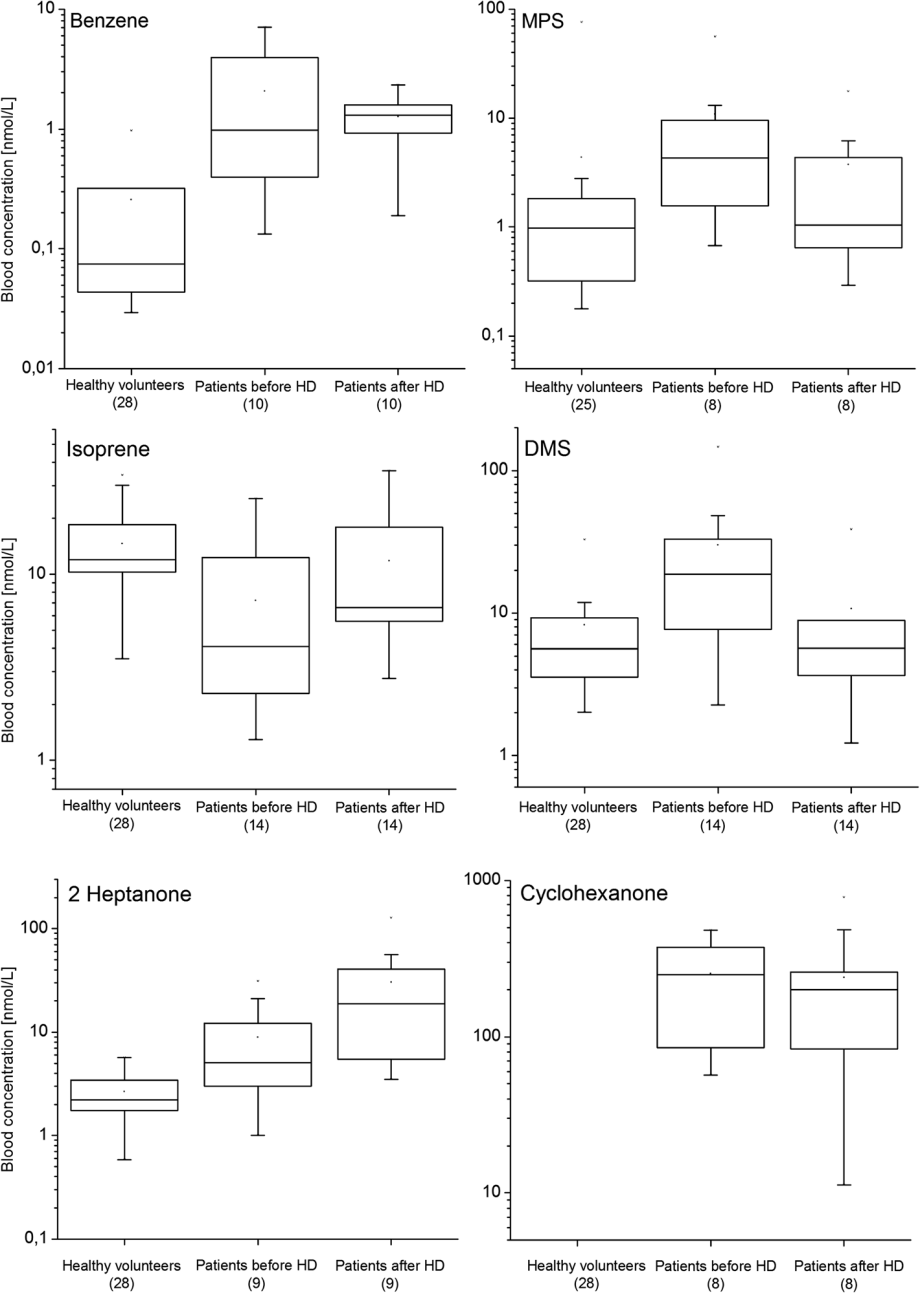
**Comparison of blood levels of selected species in dialyzed patients (before and after HD) and healthy volunteers.** Data for the latter were taken from [25].

22 aliphatic hydrocarbons were detected in the blood or breath of HD patients. A great majority of them (17) were pollutants originating from the dialyzer materials. Breath levels of four species (n-butane, n-pentane, 2-methyl-1-pentene, and 1-hexene) were significantly higher than room air ones and exhibited a statistically significant decrease during the treatment (see Figure 2 for exemplary profiles). This observation is in good agreement with the results reported by Lee *et al. *[24] evidencing the influx of hydrocarbon pollutants into blood from the extracorporeal circuit. Whereas Lee *et al. *[24] reported HC levels that were elevated only for a rather short period of time (20 minutes), the increased HC concentrations observed within this study were still present at the end of hemodialysis. The composition and intensity of the contaminant influx are clearly manufacturing process dependent and may differ significantly between different vendors. Strikingly, the aforementioned concentration drops were not observed in blood samples. This discrepancy can be explained by the poor blood solubility of aliphatic hydrocarbons [30] as well as the fact that the arterial blood sampling point was located at the beginning of the extracorporeal circulation before the membrane. Consequently, the samples correspond to blood degassed during pulmonary gas exchange. Thus, it seems that this excess of hydrocarbons is quickly and effectively removed by the lungs and the exposure of tissues to these pollutants is probably limited. Amongst the hydrocarbons not being treatment-related pollutants there were four smoking-induced species (1,3-Butadiene, (E/Z)-1,3-Pentadiene and 1-heptene) and isoprene. Both blood and breath levels of isoprene increased significantly after the hemodialysis (see Figure 3 and Figure 4). In exhaled air this rise amounted to about 36%, whereas in blood an average difference of 52% was observed. This characteristic breath isoprene behavior in response to dialysis has already been reported by numerous investigators [18-29], however, this is the first time that it is confirmed by parallel blood measurements. The advanced explanations for this phenomenon include hemodynamic stress, fluctuations of respiratory variables, or activation of metabolic pathways leading to mevalonic acid synthesis [19,21-23], nevertheless, none of them seems to be adequate [18]. Isoprene has received a growing interest in the field of breath gas analysis due to the fact that it may serve as a sensitive, non-invasive indicator of several diseases in human organism [31]. Strikingly, despite this interest the source of isoprene and its function in humans are still a matter of debate [32,33]. In this context, the elucidation of isoprene evolution during hemodialysis could be valuable for understanding its function in human organism.

A total of number of eleven ketones were quantified in blood and breath samples. For 6 of them significant differences between breath and room air levels were found (Wilcoxon signed-rank test) both before and after HD. Species from this family were also found to be affected by background emission from the dialyzer fabrics. This especially applies to cyclohexanone, 2-cyclohexen-1-one, 3-heptanone, 3-buten-2-one and 2-hexanone. Due to the shortage of the literature values for arterial blood levels of ketones in humans it is difficult to evaluate the blood concentrations obtained within this study. In our recent paper [25] we established *venous* blood levels of 62 VOCs including ketones in healthy volunteers. While VOCs levels in arterial blood are significantly lower than in venous blood due to pulmonary gas exchange [34], a comparison of results obtained within this study with the ones reported in our recent paper can provide some valuable information. In general despite degassing the arterial blood levels observed within this study were much higher than the ones observed in venous blood of healthy volunteers. For example, pre-dialysis blood levels of 2-pentanone were approx. three times higher (mean: 94 vs 35 nmol/L), 2-heptanone four times higher (mean: 10.5 vs 2.7 nmol/L) and 4-heptanone sixty five times higher (mean: 54 vs 0.83 nmol/L) than in healthy individuals (see Figure 4). The same holds true for the average breath levels (e.g. 2.78 vs 0.62 ppb for 2-pentanone, 0.6 vs 0.03 ppb for 4-heptanone). Interestingly, elevated levels of ketones in ESRD patients were present already before the HD treatment. Since ketones were reported to be particularly abundant in human urine [30,35,36], this fluid seem to be an important sink of these species in human organism. Consequently, any impairment of kidney function may significantly affect the excretion of ketones and hence lead to their retention within the uremic syndrome. Two heptanone isomers (2- and 4-heptanone) exhibited high levels in blood and breath despite the fact that they were neither found room in air nor extracorporeal circuit contaminants. Moreover, their blood levels increased significantly during the hemodialysis. Such an excess of 4-heptanone in hemodialysis patients has already been reported in the literature and attributed to the metabolism of di(2-ethylhexyl) phthalate (DEHP) [37]- a plasticizer used in polyvinyl chloride products. In humans DEHP is rapidly metabolized to 2-ethylhexanol, which is then oxidized to 2-ethylhexanoic acid and finally to 2-heptanone and 4-heptanone [37,38]. Indeed, the bloodlines used within this study were found to emit significant amounts of 2-ethylhexanol and its oxidation product 2-ethyl-hexanal. Consequently, it is plausible that the high concentrations of 2- and 4-heptanone in HD patients originated from this source. At least one of aforementioned ketones can be considered as a uremic toxin: cyclohexanone emitted from extracorporeal circuits was reported to impair cardiovascular function [39].

Only three aldehydes (2-propenal, propanal and 2-metyl-2-propenal) were found in blood samples. 2-propenal and propanal showed incidence rates of 50% and 2-metyl-2-propenal was detected only once. In breath all three species were omnipresent, however, their levels were lower (propanal), or comparable (2-propenal and 2-metyl-2-propenal) to the ones in room air. The high room air levels and the observed emission by bloodlines render the aforementioned species pollutants rather than endogenous compounds. 2-propenal was also reported to accumulate in blood of renal failure patients as the result of spermine degradation by polyamine oxidase being released from damaged kidney [40]. Since this compound was also found to be a uremic toxin damaging the functions of cells and proteins [40] its additional exogenous influx may accelerate the progression of uremia. Interestingly, while the dialyzer bloodlines also emitted several heavier aldehydes (pentanal, hexanal, heptanal, benzaldehyde, 2-ethyl-hexanal and nonanal), the latter were not detected in blood. Perhaps, the analysis of species from this chemical class requires a special sample treatment (e.g., derivatisation) [41], or they are rapidly metabolized by human tissues [42,43]. Moreover, room air concentrations of these aldehydes were generally much higher than in breath (data not shown), suggesting an additional influx of these analytes from the surrounding atmosphere.

Amongst terpenes α-pinene and 3-carene were contaminants from fabrics showing relatively high occurrences and levels in HD patients as compared to healthy volunteers [25]. p-cymene and DL-limonene also displayed very high abundances in blood, usually 20-30 times higher than in healthy individuals. However, it is not clear if they result from renal impairment, or stems from relatively high room air levels of these compounds.

Amongst aromatics there were two dialyzer pollutants (toluene and p-xylene), benzene and o-xylene. The levels of toluene tended to decrease over the course of dialysis both in blood and breath, whereas p-xylene showed stable concentrations. Benzene decreased in both fluids after haemodialysis, perhaps due to a treatment-related washout that would be consistent with the findings reported by Goerl *et al. *[29] (see Figure 3 and Figure 4). Since only 3 patients were smokers the elevated levels of benzene seem to stem from an environmental exposure. If so, this finding would evidence the retention of benzene within the uremic syndrome.

Amongst heterocyclic species only furan exhibited a difference between pre- and post-dialysis levels. Nevertheless, this effect can be ascribed to the excretion of this compound in lungs in response to its emission by bloodlines. Several heterocyclic compounds, although not detected in blood of healthy individuals [25], were quite abundant in blood of HD patients. Pyrimidine (found in 65% of all samples) showed concentration levels around 45 nmol/L, pyridine (found in 70% of all samples) around 28.6 (before HD) and 22.6 nmol/L (after HD) and 2-pentyl-furan (found in 35% of all samples) exhibited mean concentration values of 13 nmol/L. The elevated pyrimidine levels are consistent with previous studies reporting an accumulation of pyrimidine compounds in blood of patients with CKD [44].

## Conclusions

Breath and blood patterns of VOCs in dialyzed patients are notably affected by the influx of contaminants from the dialyzer materials and hospital room air. Of 60 compounds quantified in both fluids 31 were found to be emitted by extracorporeal circuit involved in the treatment. The identified pollutants belong to numerous chemical classes, however, hydrocarbons, aldehydes and ketones hold a distinguished status in this context. More volatile and poorly soluble compounds seem to be relatively quickly eliminated during pulmonary gas exchange. Exposure to highly soluble species seems to be more persistent. At least two contaminants (2-propenal and cyclohexanone) have already been identified as uremic toxins. Perhaps some other volatiles reported here are biochemically active and have negative impact on human body functions.

Excluding pollutants six volatiles tended to change their blood, or breath levels in response to the HD treatment. Only isoprene increased its concentration over the course of the hemodialysis. This finding agrees with earlier reports, however, the results within this study for the first time confirm an analogous behavior in blood. All sulphur volatiles quantified within this study decreased in concentration with dialysis time. It is not clear whether this drop stems from an effective removal of these species by the dialyzer, from changes in their endogenous production, or both. As such, additional studies are required to investigate if these species could be used as markers of hemodialysis efficiency. A concentration drop was also noted for benzene. Since an endogenous production of this compound is rather unlikely, its levels and behavior during HD may reflect previous exogenous exposure and/or reduced renal excretion and subsequent washout during the treatment.

## Competing interests

The authors declare that they have no competing interests.

## Authors’ contributions

PM contributed to the design of the study, developed the GC-MS measurement protocol, performed GC-MS analyses, calibration and validation measurements, data processing, data interpretation and wrote the draft of the manuscript. MH performed patients' interviews and documentation and collection of samples, AA and GM designed the study, supervised the experiments, revised and approved the manuscript. JK and KU performed the data analysis, contributed to their interpretation and revised the manuscript. All authors read and approved the final manuscript.

## Pre-publication history

The pre-publication history for this paper can be accessed here:

http://www.biomedcentral.com/1471-2369/15/43/prepub
